# Mineral Waters

**Published:** 1884-10

**Authors:** 


### MINERAL WATERS.

NATURAL Mineral Springs have, for ages, been regarded as possessing unequaled curative properties for certain diseases. Such marvelous stories have been told of the cures effected, that some have attributed to them supernatural power, while others have gone to the other extreme of skepticism, and profess to believe nothing of all that is claimed. Modern science and investigation have got at the truth of these claims, and there is no natural mineral spring of note, in the civilized world, whose waters have not been carefully analyzed, and their value as curative agents, accurately defined; and the diseases to which they are applicable, mentioned, with the quantities of water required.

The properties of mineral waters depend upon the character of the soil through which they percolate. Rain water is a powerful solvent, and, as it filters through the ground, dissolves some of the substances met with in its passage. Thus, if it is impregnated with some salt of iron, it is called "Chalybeate" water, and is useful as a tonic. Alkaline waters are useful in certain unnatural conditions of the system, and saline waters in others. Sulphur springs ordinarily contain iron and other remedial agents, but as sulphur is always the most striking characteristic, such waters are usually known as sulphur springs, and the most marked cases of recovery from disease generally occur at such springs. I have seen the most happy results follow the use. for a few days, of the waters of the Hot Springs of the Cauquenies in Chile. I have seen persons who had long been sufferers from gout, rheumatism. and various forms of skin diseases, return from the Hot Springs of Arkansas, after an absence of a few weeks, to all appearance quite restored to health; but I did not know, until last summer, that just as good results could be obtained at Richfield Springs—one of the most delightful spots in the State of New York,—without being compelled to encounter the fatigue and hardships that are unavoidable in reaching these remote places, and the wretched accommodations that patients have to put up with, while they remain.

I was informed that there were ten thousand people at the various hotels, boarding houses and cottages of Richfield Springs last summer. The leading hotels are the Springs House, and the New American; each has accommodations for more than five hundred persons, and the fare and accommodations are unexcelled.

The water is very strongly impregnated with sulphur, and has a wide reputation in the cure of dyspepsia, rheumatism and gout. I saw a number of persons at the Springs House who seemed to be in the enjoyment of excellent health, but who informed me that they came a few weeks before in a crippled condition and great sufferers from rheumatism; and who attributed their recovery to the baths, and to drinking the water. There were also dyspeptics who were enjoying immunity from their sufferings, through drinking the water two or three times a day, hot. But I have no doubt that the excursions on the beautiful little lakes that dot the scenery around Richfield Springs, and the rambles and drives and many other sources of enjoyment that abound there, contributed largely to the almost uniformly favorable results of the treatment. There was one feature at Richfield Springs that was "conspicuous by its absence," and that was the tendency to divide up into cliques and coteries, for the purpose of rudely monopolizing the various pleasures provided for *all* the guests. At some summer hotels, these cliques claim the choice of seats at the tables, and the *exclusive* use of the parlors for dancing and other amusements

I had an excellent excuse for a sojourn at Richfield Springs last summer (for I was ill), but I came home in such perfect health—which I have since continued to enjoy—that my anticipated visit next summer will, I trust, be simply one of recreation. To breathe. for a few weeks, the pure air, at an elevation of nearly two thousand feet above the sea, is the best and most permanent of all tonics.

### BRIGHT'S DISEASE.

ON THIS Subject, Dr. Alex. De Borra, of Crystal Springs. N. Y., writes to the *Scientific American*, that, after years of practical test of the milk diet for Bright's disease, he has a long list of cases in which he has made perfect cures. Great care is taken to get absolutely pure skimmed milk from healthy and well-fed cows. and no other food of any kind is given after the patient can bear five pints of milk a day. Up to this point, and until the stomach is able to take care of so much, is found to be the most trying period in this treatment; but no other medicine is given, and hand and hair-glove rubbing is daily administered.

A correspondent takes exception to the claim made, that no drug of any therapeutic value in that disease has yet been discovered, and gives a recipe which, he claims, has affected cures in Bright's disease as well as in dropsy, during fifteen years: "Take a double handful of the dry pods of the common white soup bean or corn bean, to three quarts of water; boil slowly for three hours, until it is reduced to three pints. Take hot or cold. Use no other drink

